# 1796 – *An Introduction to Botany*: The critical role of women in eighteenth-century science popularisation and the early promotion of science for young girls in Britain

**DOI:** 10.1177/09636625231217015

**Published:** 2023-12-14

**Authors:** Isabel Richards

**Affiliations:** Australian National University, Australia

## 1. Production and purpose of a children’s botanical text

Priscilla Wakefield’s (1796) *An Introduction to Botany: In a Series of Familiar Letters, with Illustrative Engravings* (referred to as *An Introduction* hereafter) was a late eighteenth-century children’s education book and critical milestone in an untold story of women in early Western science communication. First published in 1796 Britain, the book offers a clear and simple introduction to the Linnaean system through 27 letters between 2 fictional teenage sisters. This is accompanied by intricate sketches and descriptions in an Appendix. The main character, Felicia, is a young girl who learns about botany classifications from her governess to cheer her up while her sister, Constance, spends the summer away. Over several months, Felicia shares her lessons and growing understanding of plant science with Constance.

*An Introduction* was one of the earliest adaptions of botany for the budding market of juvenile publishing (Shteir, 1985). Writing to support her family financially after the failure of her husband’s business, Wakefield spotted a gap in the market and became the first female author of children’s scientific books (Knellwolf and Goodall, 2008; Le-May Sheffield, 2004). She was one of many English writers who began demanding a broader life for women (Peters, 2017). *An Introduction* was her second book and one that gives an insight into the level of science education deemed appropriate for young girls towards the end of the eighteenth century (George, 2006). In the preface, she writes:

May it become a substitute for some of the trifling, not to say pernicious, objects that too frequently occupy the leisure of young ladies of fashionable manners, and by employing their faculties rationally, act as an antidote to levity and idleness (Wakefield, 1796: v).

Wakefield’s Felicia is educated through many books, but a greater emphasis is put on direct observation and handling of specimens rather than purely reading about them. In her first letter to Constance, Felicia explains that ‘books should not be depended upon alone, recourse must be had to the natural specimens growing in fields and gardens’ (Wakefield, 1796: 2). By offering her child characters a degree of independence, Wakefield encourages children’s natural curiosity and inspires her young readers to be more than just passive pupils. She recognises that ‘children are endowed with curiosity and activity, for the purpose of acquiring knowledge. Let us avail ourselves of these natural propensities’ (Wakefield, 1796: iii). *An Introduction* shows how the learner, Felicia, becomes the teacher as she communicates her newly acquired scientific knowledge to her sister and develops her own methods of observation.

Coming from a prosperous London Quaker family, Wakefield managed to avoid the secularisation of science (Bensaude-Vincent, 2001; Cunningham and Williams, 1993; Peters, 2017). She promoted natural history as an entertaining way for children to strengthen their relationship with God and understand his creation of the physical world. Her emphasis on observation and reflection stemmed greatly from these Quaker affiliations (Knellwolf and Goodall, 2008). For Quakers, hands-on, experimental science was prized because it revealed God’s eternal purposes while avoiding the indolence of contemplation (Leach, 2006). Wakefield (1796) explicitly states her intentions to ‘cultivate a taste in young persons for the study of nature, which is the most familiar means of introducing suitable ideas of the attributes of the Divine Being’ (p. iii).

## 2. *An Introduction* as an exemplar of late eighteenth-century British science popularisation

During the late eighteenth century to early nineteenth century, popular science books played a key role in women’s education (Rossiter, 1982). Though these texts were commonly written by females, the history of women in popular science is usually considered separate to the history of popular science (which is disproportionately focused on male authors) (Govoni, 2009). This distinct separation has hindered progress in our research of the history of science and science communication. It is pertinent, then, that we begin looking at female author contributions throughout history as exemplars of science popularisation to establish a more holistic understanding of science communication.

In this section, I argue that *An Introduction* is a typical example of science popularisation during the European Enlightenment. The text is welcoming of a wide audience because it is jargon-free, affordable and entertaining. It is also associated with Enlightenment philosophies, including botany as an everyday activity for both men and women. In addition, the book was well-received by the community through several reviews, reprints and translations.

*An Introduction* is standard eighteenth-century science popularisation because of its accessibility. The text is accessible to its intended child audience, and the wider public, in many ways. First, the book is reasonably priced at 4*s*. 6*d*. (the equivalent of $27 AUD) and intentionally minimises jargon (The Quarterly Review, 1832). The letter format forces a short and succinct recount of plant anatomy. Wakefield (1796) claims in her preface that ‘everything hitherto published was too expensive, as well as too diffuse and scientific, for the purpose of teaching the elementary parts to children’ (p. v).

Not only is the book inexpensive, but also lively and entertaining. Wakefield draws on literary devices and discusses the real-world applications of plants to engage readers, while also providing illustrations pleasing to the eye. These techniques are defining features of eighteenth-century British science writing, where pleasant, expressive prose was favoured over complicated, verbose descriptions to enhance the reading experience (Fontes da Costa, 2009; Montgomery, 1996). It was particularly typical of female scientific authors to adopt a personal style with fictional dialogues, placing women instead of men in the position of authority (Knellwolf and Goodall, 2008). The main literary devices she uses are metaphors and similes, comparing plant parts to human anatomy. For example, Felicia describes roots as ‘many mouths’ that ‘absorb the nutritious juices from the earth’ (Wakefield, 1796: 4) and leaves as ‘lungs’ that ‘by their inclination to be moved by the wind, in some degree, serve also as those of muscles’ (Wakefield, 1796: 6). Wakefield also simplifies plant classifications by comparing them with human divisions. In her sixth letter, Felicia writes that ‘Vegetables resemble Man; Classes, Nation of Men; Orders, Tribes, or Divisions of Nations; Genera, the Families that compose the Tribes; Species, Individuals of which Families consist; Varieties, Individuals under different appearances’ (Wakefield, 1796: 29).

**Figure 1.**
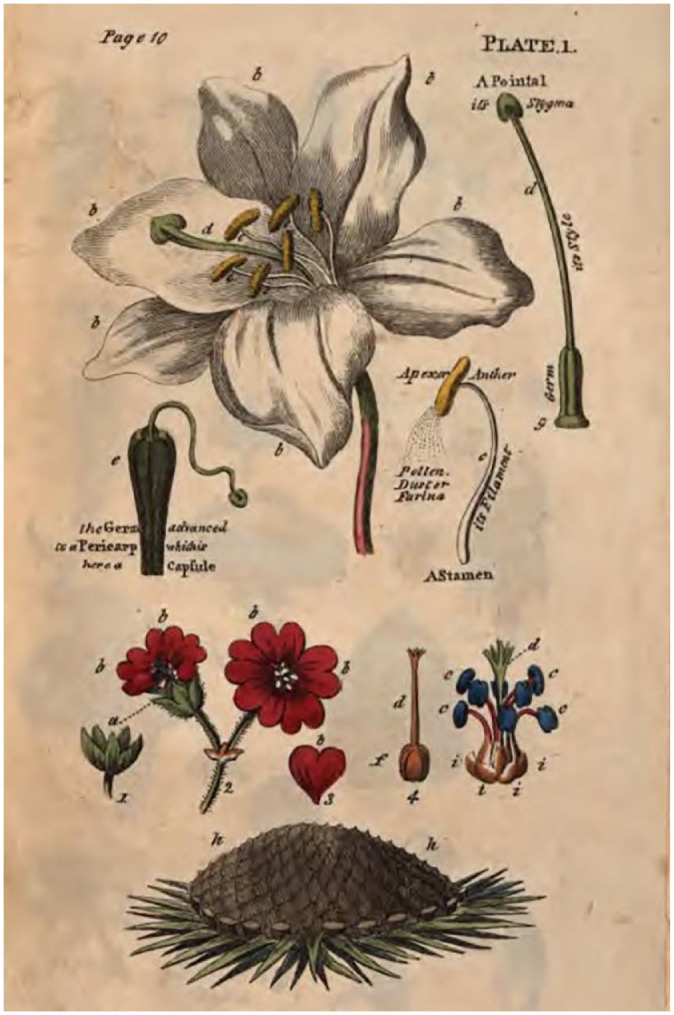
Parts of flowers as outlined in letter III of Wakefield’s *An Introduction.*

By comparing plants with people, it makes them less dull and more relatable and grants young readers a clearer understanding of vegetation functions. This poetic style of writing adds to the wonder of nature and nourishes a child’s curiosity (Fontes da Costa, 2009). The book’s sketches complement this, adding a visual reference to Wakefield’s descriptions and making them come to life. An example of this can be seen in Figure 1.

Thus, the book is an exemplar of eighteenth-century British science popularisation because of its accessibility to a wide audience, allowing Wakefield to develop a close relationship with her readers.

*An Introduction* can also be deemed traditional eighteenth-century science popularisation as its attitudes and intentions align with Enlightenment philosophies. Wakefield’s campaign for science as an everyday activity, portrayals of botany and views on female education are linked to typical Enlightenment ideas. Wakefield’s promotion of regular individual scientific exploration is directly related to the Enlightenment encouragement of amateur science, rationality and knowledge growth. During the eighteenth century, science experiments were performed in both private, elite settings and small, amateur laboratories (Bensaude-Vincent, 2001; Lynn, 2006). In Britain, botany publications tended to blur the lines between scientists and laypersons, both groups improving each other’s knowledge (Secord, 1994). Likewise, Wakefield treats science as an everyday activity that takes place at home and fosters personal observation through common household appliances rather than exclusive equipment. Felicia recommends to Constance that ‘to assist you in the examination . . . it will be necessary to provide a magnifying glass, a needle, lancet, and a pair of small scissors’ (Wakefield, 1796: 23). At the time of *An Introductions’s* publication, observation and critical thinking based on reasoning in amateurs were respected (Bensaude-Vincent, 2001; Lynn, 2006). *An Introduction* was published shortly after Immanuel Kant’s (1784) *What is Enlightenment?*, sharing its goals of urging people to use reason to think for themselves and no longer rely on experts. Wakefield recognises the many exceptions to rules when classifying plants, advising readers to reach a verdict using common sense and their natural observational skills. For example, Felicia teaches Constance to ‘always examine the contents of the flower carefully . . . as there are plants of a different construction, that resemble these in appearance, at least to the eye of a superficial observer’ (Wakefield, 1796: 71).

In terms of botany, a knowledge of plants and their uses was seen as important for introducing new European commodities and maintaining good health (Raj, 2013). In *An Introduction*, Wakefield’s (1796) Felicia regularly makes a point of acknowledging plant applications:

We must not confine our information to the form of plants, or the number of their parts, but should extend our researches to the purposes to which they are applied; a study that will supply us with much useful knowledge and entertainment (pp. 84–85).

She then goes on to discuss their various benefits for medicine, cooking and dyeing. Furthermore, popular botany in Britain was considered a way for individuals to increase their appreciation and love of plants (Secord, 2002; Topham, 2009). Botany was defined as an observational classificatory science, where images provided sensory enjoyment (Secord, 2002). This is reflected in *An Introduction* through Wakefield’s (1796) Quaker affiliations and focus on the beauty of nature as well as her use of pictures, as discussed earlier. Felicia reminds Constance to ‘use your eyes, and let none of Flora’s beauties escape your observation’ (p. 77).

In terms of female education, Wakefield held a strong belief in equality of elementary schooling for boys and girls (Peters, 2017). She argued that women possessed the same abilities as men but had not been taught how to access them (Peters, 2017). She also felt that married women should have scientific knowledge, so they could manage domestic affairs, finances and educate their children (Hill, 1997; Knellwolf and Goodall, 2008; Peters, 2017). However, she did warn women that excessive attention to scientific education would detract from domestic duties (Peters, 2017). For all these reasons, she wrote *An Introduction*. These beliefs match well with late eighteenth-century to early nineteenth-century views on involving women in botany as gatekeepers of knowledge and its circulation, with works specifically targeted at a wealthy female audience (Bensaude-Vincent, 2001; Fontes da Costa, 2009; Secord et al., 1996). The text is exemplary of botany feminisation because it offers women access to scientific knowledge while still retaining conservative function (George, 2006). Thus, the book is standard eighteenth-century science popularisation due to its alignment with Enlightenment philosophies of amateur science, botanical depictions and female education.

Moreover, *An Introduction* is a typical example of eighteenth-century science popularisation because of its popular reception. Botanical science rose to popularity in late eighteenth-century Britain because of wide-ranging interest in exotic discoveries brought back from the ‘New World’ (Montgomery, 1996). Consequently, Wakefield’s book was translated into French in 1801, widely circulated through 11 editions up until 1841 and reviewed by the British and French critical press as well as academic journals (George, 2006; Martin, 2011). In the later editions, Sarah Hoare’s Darwin-inspired *Poem on the Pleasures and Advantages of Botanical Pursuits* was appended (George, 2006). This was typical of female authors, who aimed to combine the popularisation of knowledge with aesthetic pleasure and the improvement of moral virtues (Fontes da Costa, 2009; Shteir, 1996). This extensive acceptance is yet another example of how *An Introduction* is consistent with the conventions of science popularisation in eighteenth-century Britain.

## 3. Conclusion

Priscilla Wakefield’s *An Introduction* is a significant historical item that encourages rationality and children’s natural curiosity (particularly that of young girls) to understand the wonders of the physical world, filling a niche market in the publishing of children’s scientific books and highlighting the important, though often overlooked, role of women in early Western science communication. The text is clearly an exemplar of late eighteenth-century science popularisation because of its accessibility, alignment with eighteenth-century philosophies and overall popularity. Future studies should investigate how other eighteenth-century popular science texts written by females – particularly those published outside of Britain – align with science popularisation theories for a more holistic understanding of historical Western science communication.
